# Resveratrol Alleviates Inflammatory Response Through P2X7/NLRP3 Signaling Pathway: In Silico and In Vitro Evidence from Activated Microglia

**DOI:** 10.3390/ph18070950

**Published:** 2025-06-24

**Authors:** Bianca Fagan Bissacotti, Marcylene Vieira da Silveira, Charles Elias Assmann, Priscila Marquezan Copetti, André Flores dos Santos, Solange Binotto Fagan, João Augusto Pereira da Rocha, Maria Rosa Chitolina Schetinger, Vera Maria Melchiors Morsch, Nathieli Bianchin Bottari, Alencar Kolinski Machado, Aleksandro Schafer da Silva

**Affiliations:** 1 Graduate Program in Biological Sciences: Toxicological Biochemistry, Department of Biochemistry and Molecular Biology, Federal University of Santa Maria (UFSM), Santa Maria 97105-900, RS, Brazil; bianca_fbissacotti@hotmail.com (B.F.B.); marcyvieirass@outlook.com (M.V.d.S.); charles.ufsm@gmail.com (C.E.A.); priscilaa_marquezan@hotmail.com (P.M.C.); mariachitolina@gmail.com (M.R.C.S.); veramorsch@gmail.com (V.M.M.M.); 2 Graduate Program in Nanosciences, Franciscan University (UFN), Santa Maria 97010-032, RS, Brazil; andreflores2009@gmail.com (A.F.d.S.); solange.fagan@gmail.com (S.B.F.); alencarkolinski@gmail.com (A.K.M.); 3 Laboratory of Modeling and Computational Chemistry, Federal Institute of Education, Science and Technology of Pará (IFPA), Bragança Campus, Bragança 68600-000, PA, Brazil; joao.rocha@ifpa.edu.br; 4 Department of Microbiology and Parasitology, Federal University of Pelotas (UFPel), Pelotas 96010-610, RS, Brazil; nathieli_bb@hotmail.com; 5 Graduate Program in Animal Science, Santa Catarina State University (UDESC), Chapecó 89815-630, SC, Brazil

**Keywords:** brain inflammation, BV-2 cells, immunomodulation, polyphenol, purinergic receptors

## Abstract

**Background/Objectives**: Chronic inflammation and inappropriate NLRP3 inflammasome regulation are related to many brain diseases. Purinergic mediators may play an important role in inflammation regulation and could be targeted for effective therapies for these illnesses. We evaluated resveratrol’s anti-neuroinflammatory potential in BV-2 microglia cells using an innovative in vitro method of NLRP3 inflammasome activation, correlating with the P2X7 purinergic receptor. **Methods**: In silico analyses were used to estimate resveratrol’s interaction with NLRP3, and its cytotoxicity was measured for 24, 48, and 72 h. Moreover, microglia were exposed to lipopolysaccharide and nigericin to activate the NLRP3 inflammasome and treated with resveratrol between these inflammatory agents. **Results**: It was found that resveratrol has binding compatible with modulating NLRP3. Specifically, 0.1–25 µM of resveratrol presented a favorable safety profile in BV-2 cells. Microglia exposed to the inflammatory agents had increased levels of oxidative species, the P2X7 receptor, and pro-inflammatory cytokines. However, resveratrol decreased the NLRP3, caspase-1, IL-1β, IL-6, and TNF-α mRNA levels and protein density; on the other hand, IL-10 was increased, acting as a protector, preventing exacerbated inflammation. Under resveratrol exposure, P2X7 was negatively expressed, regulating inflammation to establish homeostasis and microglial proliferation. Additionally, resveratrol activates the A1 adenosine receptor, possibly correlated with neuroprotective effects. **Conclusions**: We confirmed the anti-neuroinflammatory action of resveratrol via the P2X7 receptor and NLRP3’s combined modulation, regulating the cell cycle and reducing pro-inflammatory and oxidant agents. Considering this pathway, resveratrol could be a candidate for further investigations as a potential treatment against neuroinflammatory diseases.

## 1. Introduction

Inflammation is a natural organism’s protective response against pathogens and stressors [1]. In the central nervous system (CNS), microglia and astrocytes are the primary immune defense cells, which can express the proteins that form the NOD-like receptor family pyrin domain-containing 3 (NLRP3) inflammasome and activate it [1,2,3].

The NLRP3 inflammasome is a multiprotein complex formed by the proteins NLRP3 and pro-caspase-1 and the apoptosis-associated adapter protein containing the CARD domain (ASC) [2]. The union and activation of these molecules involve the presence of pathogens (viruses, bacteria, parasites, fungi) or tissue damage (reactive oxygen or nitrogen species release, ROS and RNS, respectively). Immediately after NLRP3 activation, active caspase-1 (CASP-1) is released, followed by interleukin-1 beta (IL-1β) and interleukin-18 (IL-18), resulting in the subsequent pro-inflammatory cascade [2,3,4,5]. The priming and activation mechanisms are the primary regulators of NLRP3. Priming occurs through recognizing pathogen-associated molecular patterns (PAMPs), such as lipopolysaccharide (LPS) found in some bacteria, or damage-associated patterns (DAMPs), which increase the mRNA transcription of inflammatory components [6,7]. The entire NLRP3 activation process comprises assembly and activation steps. Decreases in the intracellular potassium and calcium ion levels and the production of ROS can act as activation signals. Nigericin (NIG) leads to the induction of potassium efflux, a potent activator of the NLRP3 inflammasome [7,8].

It is essential to mention that the purinergic system is an expressive contributor to the immune response and the NLRP3 inflammasome’s activation. The purinergic pathway involves receptors and molecules that act as extracellular signals to modulate many processes in the body [9,10]. When activated/expressed, the purinergic receptors P2X7 (which binds adenosine triphosphate) and A1 (which binds adenosine) are responsible for developing inflammatory and neuroprotective responses, respectively [11,12,13]. Interestingly, there are some studies involving psychiatric and neurodegenerative diseases, as well as neurotoxoplasmosis, that show a strong relationship between the P2X7-NLRP3-IL-1β pathway and the illness’s pathophysiology, mainly in terms of the inflammatory response [14,15,16,17,18]. Despite the A1 receptor being able to attenuate the inflammatory response and inhibit the activation of immune cells [13,19], there is still a lack of knowledge of the A1 receptor’s involvement within the NLRP3 inflammasome and P2X7 receptor context.

Chronic inflammation and inappropriate NLRP3 inflammasome regulation in the brain have been associated with degenerative, psychiatric, and parasitic diseases, such as Alzheimer’s disease, Parkinson’s disease, schizophrenia, bipolar disorder, major depression, and neurotoxoplasmosis [20,21,22,23,24]. These diseases have complex pathophysiologies and are considered increasingly common public health problems worldwide [25,26]. Additionally, treating these illnesses is very difficult, and many research groups look forward to the development of scientific investigations into new possible therapies for these diseases. MCC950 is an example of a molecule that has been studied to attenuate inflammation; however, this agent can present toxicity by inhibiting NLRP3 completely [27].

On the other hand, natural health products are being studied, considering their medicinal effects, associated with antioxidant and anti-inflammatory actions [28,29]. Resveratrol (RSV) is a natural polyphenol, found in the skins and seeds of grapes, blueberries, blackberries, and peanuts, that has gained scientific attention [30,31,32]. This polyphenol has pleiotropic action, mediated by multiple molecular pathways, showing remarkable biological properties and potential therapeutic benefits in several experimental models, as demonstrated in a review study [30]. These multiple properties of RSV are of interest in conditions where oxidative stress, dysregulated inflammation, and excessive cell death play a central role in the pathophysiology, as observed in neurodegenerative diseases and inflammation in microglial cells, for example [30,31,32,33].

RSV has demonstrated the ability to act as a potent antioxidant, both through the direct scavenging of ROS and through the modulation and induction of antioxidant enzymes, such as superoxide dismutase and catalase, often via the activation of the nuclear factor erythroid 2-related factor 2 pathway, in 3D neuronal culture in pro-inflammatory conditions [30,34]. Its anti-apoptotic action was observed in cellular protection against programmed death, mediating the expression of Bcl-2 proteins and caspase activity in a study with rats exposed to a pesticide and treated with RSV [35], promoting cellular survival and homeostasis [30,31]. In addition, RSV exerts significant anti-inflammatory actions, inhibiting the activation of pro-inflammatory pathways such as nuclear factor kappa B and the consequent reduction in pro-inflammatory cytokine production and modulating the expression of enzymes such as COX-2 and iNOS [30,33]. Notably, RSV has also been associated with NLRP3 inflammasome and purinergic signaling modulation (the targets of this study), suggesting its therapeutic potential through controlling chronic inflammation and the consequent complications [33,34,36,37,38,39].

Despite this, the proof of these modulations using a methodology considering the NLRP3 inflammasome’s activation has not been considered. Therefore, we aimed to evaluate the anti-neuroinflammatory potential of RSV through in silico methods and via an innovative in vitro method of NLRP3 inflammasome activation (examining the preventive effect of RSV on the inhibition of inflammasome protein oligomerization) in microglia cells (BV-2 cell line), with a particular focus on the pathway involving the A1 and P2X7 purinergic receptors.

## 2. Results

### 2.1. RSV Has Binding Affinity for the PYD Domain of NLRP3

An in silico analysis was performed to evaluate the interaction of RSV with the PYD region of NLRP3 and compare it with the MCC950 inhibitor. As expected, with data obtained by molecular docking, the best target affinity result was for MCC950 (−8.4 kcal/mol). For RSV, the calculated value was −6.7 kcal/mol. The concentration of 12.27 µM of RSV was obtained in our results as capable of inhibiting half of the NLRP3 activity. However, for MCC950, the estimated Ki was 696.25 nM. The root mean square deviation (RMSD) values were 0.480 Å for RSV and 0.541 Å for MCC950, compatible with target binding (Table 1).

**Figure 1:**
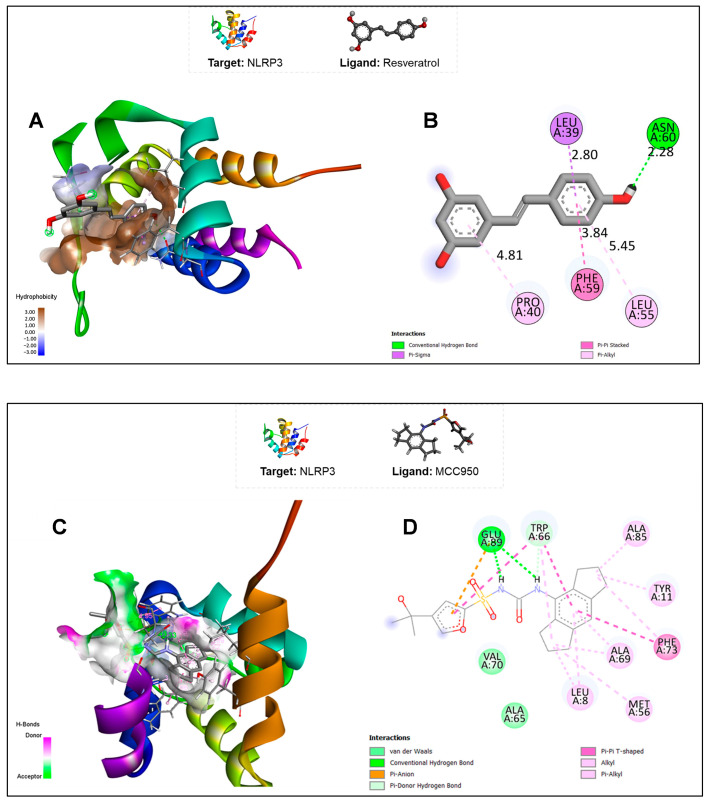
(**A**) Interactions between the PYD domain of the NLRP3 protein and RSV, highlighting its hydrophobicity; (**B**) 2D map of interactions between the PYD domain of the NLRP3 protein and RSV, highlighting the amino acid residues that participate in the bonds and the types of bonds; (**C**) interactions between the PYD domain of the NLRP3 protein and MCC950, highlighting the regions of hydrogen bond formation; (**D**) 2D map of interactions between the PYD domain of the NLRP3 protein and MCC950, highlighting the amino acids that participate in the bonds and the types of bonds.

Figure 1 demonstrates the primary conformation possibility and the bond types between the RSV (Figure 1A,B) or MCC950 (Figure 1C,D) atoms and the PYD target domain of NLRP3. Highlighting RSV, the existing bonds with the NLRP3 inflammasome are hydrogen bonds, Pi–Sigma, stacked Pi–Pi, and Pi–alkyl, with the amino acids leucine (A:39), asparagine (A:60), leucine (A:55), phenylalanine (A:59), and proline (A:40) (Figure 1B).

**Figure 2:**
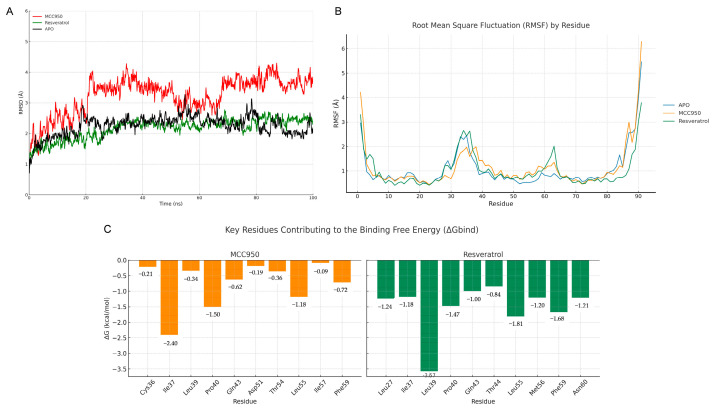
(**A**) Root mean square deviation (RMSD) of the simulations for the APO, MCC950, and RSV systems; (**B**) root mean square fluctuation (RMSF) per residue for the APO, MCC950, and RSV systems; (**C**) per-residue binding free energy contributions of MCC950 and RSV to the PYD domain (PDB ID: 2NAQ).

Molecular dynamics simulations were carried out to evaluate the structural stability of the complexes formed between the PYD domain of NLRP3 (PDB ID: 2NAQ) and the ligands MCC950 and RSV. The APO form was also simulated as a comparative control. An analysis of the RMSD revealed that the APO system maintained average stability of 2.26 Å (±0.33 Å), while the RSV complex exhibited the lowest average RMSD of 2.17 Å (±0.33 Å), indicating the favorable accommodation of the ligand within the binding site. In contrast, the MCC950 complex showed a higher average RMSD of 3.25 Å (±0.60 Å), suggesting greater conformational flexibility throughout the simulation (Figure 2A; Table 2).

Root mean square fluctuation (RMSF) analysis revealed that terminal residues exhibited higher mobility, as expected. However, differences were also observed in internal regions: the MCC950 complex displayed slightly higher fluctuation peaks compared to the APO form, whereas the RSV complex showed a marked reduction in the mobility of the central region of the PYD domain, suggesting a stabilizing effect (Figure 2B).

The radius of gyration (Rg) indicated distinct structural compactness among the systems: the MCC950 complex showed the most significant expansion (27.03 Å ± 2.18), followed by the APO form (22.59 Å ± 1.87), while the RSV complex exhibited the highest degree of compactness (21.55 Å ± 1.22), reinforcing its ability to restrict protein flexibility (Table 2). Binding free energy analysis using the MM/GBSA approach confirmed the higher binding affinity for RSV (−22.44 ± 5.91 kcal/mol) compared to MCC950 (−13.63 ± 11.08 kcal/mol), with significant contributions from both electrostatic and van der Waals interactions in the case of RSV (Table 3).

Finally, the energy decomposition per residue highlighted Leu39, Pro40, Leu55, and Phe59 residues as the main contributors to the binding of both ligands. RSV showed a more favorable interaction with Leu39 (ΔG = −3.57 kcal/mol), while MCC950 interacted more intensely with Ile37 (ΔG = −2.40 kcal/mol) (Figure 2C).

### 2.2. RSV Decreases Cell Viability Without Increasing the Oxidative Profile

**Figure 3:**
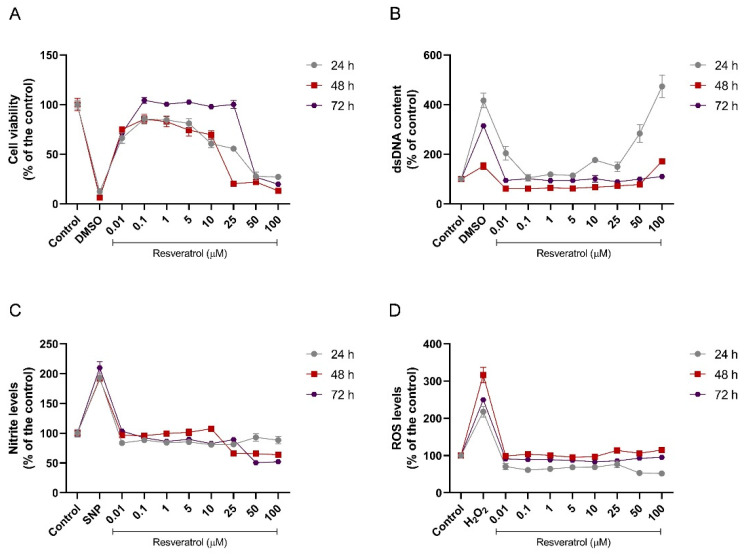
Concentration–response curve of RSV in the microglial cells (BV-2 cell line)—in vitro safety profile. (**A**) Cell viability for 24, 48, and 72 h exposure to RSV; (**B**) dsDNA free content for 24, 48, and 72 h exposure to RSV; (**C**) nitrite levels for 24, 48, and 72 h exposure to RSV; (**D**) ROS levels for 24, 48, and 72 h exposure to RSV. SNP—sodium nitroprusside. The experiments were performed in triplicate. The statistical analysis is demonstrated in Supplementary Material S1 (with the same results in a column graph) and was conducted by one-way ANOVA followed by the Tukey post hoc test. Data are expressed as mean values ± SEM.

To evaluate its safety, the murine microglia BV-2 cell line was initially exposed to a concentration curve of RSV (0.01–100 µM) for 24, 48, and 72 h (Figure 3). Figure 3 represents a concentration–response curve according to the exposure times. The statistical differences can be observed in Supplementary Material S1, were the same results are demonstrated in a column graph to facilitate their visualization.

During the 24 h incubation period, BV-2 cells showed a decrease in cell viability when exposed to concentrations of 0.01 µM (33.67% reduction, *p* < 0.0001), 10 µM (39.27% reduction, *p* < 0.0001), 25 µM (44.28% reduction, *p* < 0.0001), 50 µM (72.22% reduction, *p* < 0.0001), and 100 µM (72.75% reduction, *p* < 0.0001) when compared to the control (Figure 3A). In addition, membrane damage could be observed when testing the free dsDNA content in the supernatant. Only concentrations of 0.01 µM (*p* = 0.0065), 50 µM (*p* < 0.0001), and 100 µM (*p* < 0.0001) significantly increased this parameter (104.4%, 183.5%, 373.1%, respectively) compared to the control (Figure 3B). Increases in the oxidative profile were not observed; only a decrease in ROS was found under 0.1 µM (*p* = 0.0102), 1 µM (*p* = 0.0235), 50 µM (*p* = 0.0009), and 100 µM (*p* = 0.0005) concentrations (38.93%, 35.98%, 46.86%, and 48.28% reductions, respectively) (Figure 3C,D).

After 48 h of incubation, the decreases in cell viability were maintained under 0.01 µM of RSV (25% reduction compared to the control, *p* = 0.0011). Moreover, 5 and 10 µM of RSV decreased the cell viability to 25.65% (*p* = 0.0007) and 30.32% (*p* < 0.0001) when compared to the control, respectively. The most significant reduction was observed at 25, 50, and 100 µM of RSV (79.74%, 77.89%, and 86.80% reduction compared to the control, *p* < 0.0001). Only 100 μM of RSV increased the dsDNA levels (71.83% compared to the control, *p* < 0.0001) (Figure 3A,B). Unlike what was observed in 24 h, changes in the ROS levels were not observed; however, there was a decrease in the nitrite levels when the cells were exposed to 25, 50, and 100 µM concentrations of RSV (33.82%, 34.28%, and 36.10% reductions, respectively, when compared to the control group, *p* < 0.0001) (Figure 3C,D).

At 72 h of incubation, cell viability was reduced at 0.01 µM of RSV, repeating the other results (28.68% reduction compared to the control, *p* < 0.0001). The two highest concentrations of RSV reduced the cell viability more harmfully, presenting significance of *p* < 0.0001 (50 µM—73.11% and 100 µM—80.15% reduction in cell viability when compared to the control) (Figure 3A). Decreases in oxidative parameters were also observed at this time, at concentrations of 50 and 100 µM for nitrite (49.38%; 47.83%, respectively, *p* < 0.0001) and 10 and 25 µM for ROS (16.86%*—p* = 0.0003; 14.13%*—p* = 0.0037, respectively) (Figure 3C,D). Changes were not observed for the dsDNA release test (Figure 3B).

### 2.3. Microglia with Activated NLRP3 and Pre-Treated with RSV Arrest Cell Cycle

**Figure 4:**
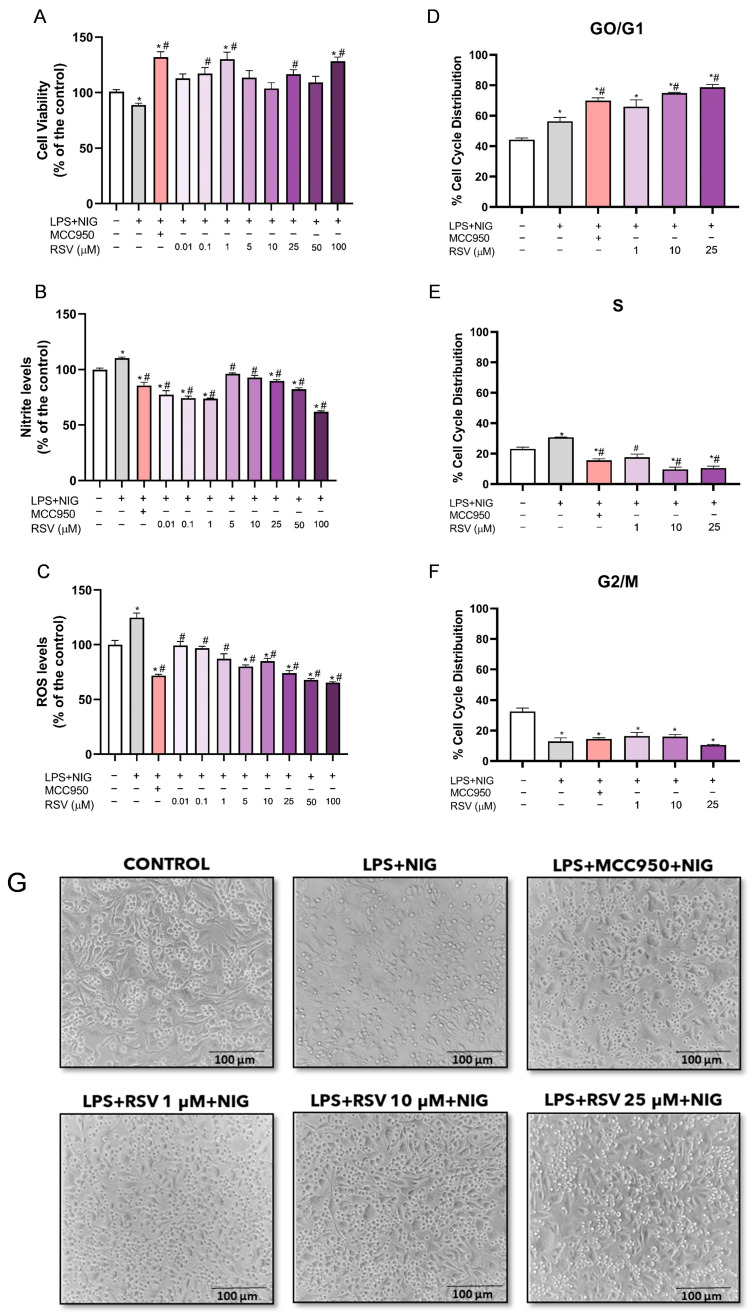
Effects of RSV on cell viability, oxidative profile, and cell cycle of BV-2 cells subjected to the NLRP3 activation protocol. (**A**) Cell viability; (**B**) nitrite levels; (**C**) ROS levels; (**D**–**F**) phases of cell cycle; (**G**) illustrative image of BV-2 cells and their respective treatments. Scale bar: 100 μm. The experiments were performed in triplicate. The statistical analysis was conducted using one-way ANOVA followed by the Tukey post hoc test. *p* < 0.05 was considered significant. Data are expressed as mean values ± SEM. * means comparison to the control group; # means comparisons to the activation group (LPS + NIG).

BV-2 microglial cells were exposed to signals to activate the NLRP3 inflammasome. As expected, cellular oxidative stress was generated for the activation control (LPS + NIG), with an increase in ROS (*p* < 0.0001) and nitrite (*p* = 0.0124) levels. Moreover, the decrease in cell viability was confirmed by the MTT test when compared to the control group (*p* = 0.013) (Figure 4A–C). MCC950, used as an inflammasome inhibitor control, reduced the ROS and nitrite levels and increased the cell viability compared to the activation control (*p* < 0.0001; *p* = 0.0001; *p* = 0.0007, respectively) (Figure 4A–C).

RSV was added to this protocol to evaluate its potential preventive effect in terms of inhibiting inflammasome protein oligomerization. RSV preventive treatment increased the cell viability when compared to the activation control at 0.1, 1, 25, and 100 µM concentrations (*p* = 0.0130; *p* < 0.0001; *p* = 0.0183; *p* < 0.0001, respectively) (Figure 4A). Additionally, all concentrations of RSV tested were able to reduce nitrite significantly, as well as the ROS levels generated in the in vitro NLRP3 inflammasome activation model, when compared to the activation control (LPS + NIG) (*p* < 0.0001, only 5 μM, *p* = 0.0002, for nitrite levels) (*p* < 0.0001 for all RSV concentrations in ROS levels) (Figure 4B,C).

Cell cycle measurement was performed to verify the cell proliferation index (Figure 4D–F). The activation control (*p* = 0.0012), the MCC950 inhibitor (*p* = 0.0021), and all concentrations of RSV (*p* = 0.0051; *p* = 0.008; *p* = 0.0004, respectively) increased the G0/G1 phase compared to the control. MCC950 (*p* = 0.0482) and 10 µM (*p* = 0.0114) and 25 µM (*p* = 0.0043) of RSV also increased this phase when compared to the activation control (LPS + NIG) (Figure 4D). However, only the activation control increased the S phase (*p* = 0.0412), while MCC950 and RSV had the opposite effect, with a decrease compared to the control group (MCC—*p* = 0.0439; 10 µM—*p* = 0.0025; 25 µM—*p* = 0.0035) and activated cell group (MCC—*p* = 0.0013; 10 µM—*p* = 0.0029; 10 µM—*p* = 0.0002; 25 µM—*p* = 0.0003) (Figure 4E). In the G2/M phase, only a decrease was observed (activation control—*p* = 0.0019; MCC—*p* = 0.0029, 1 µM—*p* = 0.0053; 10 μM—*p* = 0.0046; 25 µM—*p* = 0.0010; compared to the control group), with no difference between the activation control (LPS + NIG) and the inhibition control (MCC) and RSV preventive treatments (Figure 4F).

Figure 4G illustrates the microglia exposed to inflammasome activation conditions and RSV preventive treatment at 1, 10, and 25 µM concentrations. It is possible to observe that the group exposed to LPS + NIG presented fewer cells compared to the control. These cells also showed a different morphology (from fusiform to circular). On the other hand, when cells were treated with MCC950 or 1, 10, or 25 µM of RSV, the number of cells and morphology seemed to be at least partially recovered.

### 2.4. P2X7 and A1 Receptors Are Altered by RSV Treatment in Active Microglia

**Figure 5:**
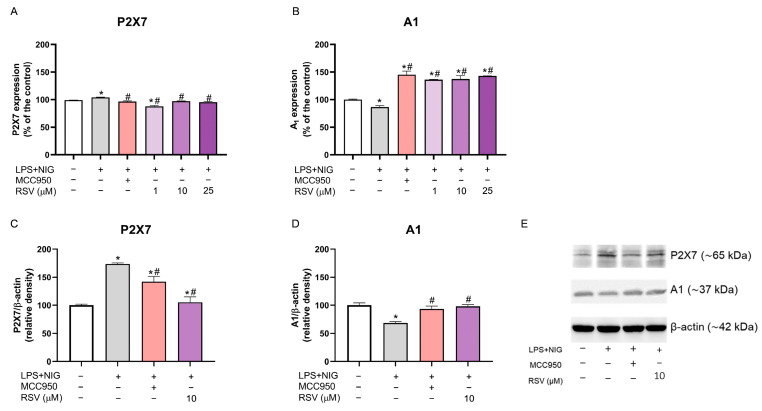
Effects of RSV on the purinergic receptor in BV-2 cells subjected to the NLRP3 activation protocol. (**A**) P2X7 expression by flow cytometer analysis; (**B**) A1 expression by flow cytometer analysis; (**C**) relative protein density of P2X7 receptor by Western blot analysis; (**D**) relative protein density of A1 receptor by Western blot analysis; (**E**) Western blot analysis showing the respective protein bands. The experiments were performed in triplicate. The statistical analysis was conducted using one-way ANOVA followed by the Tukey post hoc test. *p* < 0.05 was considered significant. Data are expressed as mean values ± SEM. * means comparison to the control group; # means comparisons to the activation group (LPS + NIG).

NLRP3 activation control increased P2X7 expression (*p* = 0.0104) and the protein density (*p* = 0.003) (compared to the control group), contributing to exacerbated inflammation and the constant formation of the NLRP3 inflammasome (Figure 5A,C). Meanwhile, the 10 and 25 μM concentrations of RSV (*p* = 0.0164; *p*= 0.0048, respectively) and MCC950 (*p* = 0.0330) restored the P2X7 expression, reducing the values when compared to the activation control (Figure 5A). Only 1 μM of RSV decreased the P2X7 levels compared to the control (*p* = 0.0013) and activation control (*p* = 0.0002) (Figure 5A). The relative protein density of P2X7 was reduced when using MCC (*p* = 0.008) and 10 μM RSV (*p* = 0.0009); the reduction is more evident in RSV compared to the activation control (Figure 5C).

A1 receptor expression and protein levels were reduced in the NLRP3 activation control (LPS + NIG; *p* = 0.0100; *p* = 0.0017, compared to the control group) (Figure 5B,D). The MCC950 treatment showed overexpression of the A1 receptor (*p* = 0.0013, compared to the control and *p* = 0.0072 compared to the activation control—LPS + NIG) (Figure 5B). Additionally, all RSV concentrations increased this receptor’s expression when compared to the control (1 µM—*p* = 0.0042; 10 μM—*p* = 0.0035; 25 µM—*p* = 0.0017) and activation control (1 µM—*p* = 0.0008; 10 μM—*p* = 0.0007; 25 µM—*p* = 0.0004) (Figure 5B). Moreover, 10 μM of RSV was sufficient to reestablish the A1 relative protein density, with no differences between the control and activation control (*p* = 0.0027) (Figure 5D). Figure 5E demonstrates the protein bands corresponding to P2X7 and A1, utilized to calculate the relative protein density (entire membranes are given in Supplementary Material S2).

### 2.5. NLRP3, CASP-1, and Cytokines Are Restored with RSV in Microglia Exposed to Inflammasome Activation Conditions

**Figure 6:**
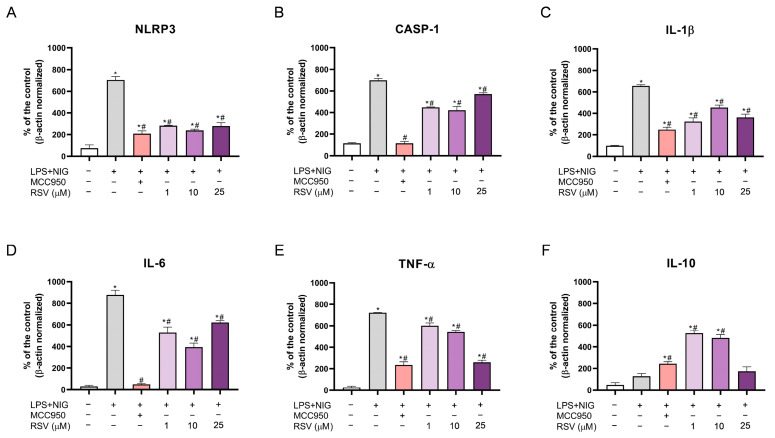
RSV modulation in the inflammatory mRNA levels in BV-2 microglial cells under exposure to NLRP3 inflammasome activation conditions. (**A**) NLRP3 mRNA levels; (**B**) caspase-1 mRNA levels; (**C**) IL-1β mRNA levels; (**D**) IL-6 mRNA levels; (**E**) TNF-α mRNA levels; (**F**) IL-10 mRNA levels. The experiments were performed in triplicate. The statistical analysis was conducted using one-way ANOVA followed by the Tukey post hoc test. *p* < 0.05 was considered significant. Data are expressed as mean values ± SEM. * means comparison to the control group; # means comparisons to the activation group (LPS + NIG).

**Figure 7:**
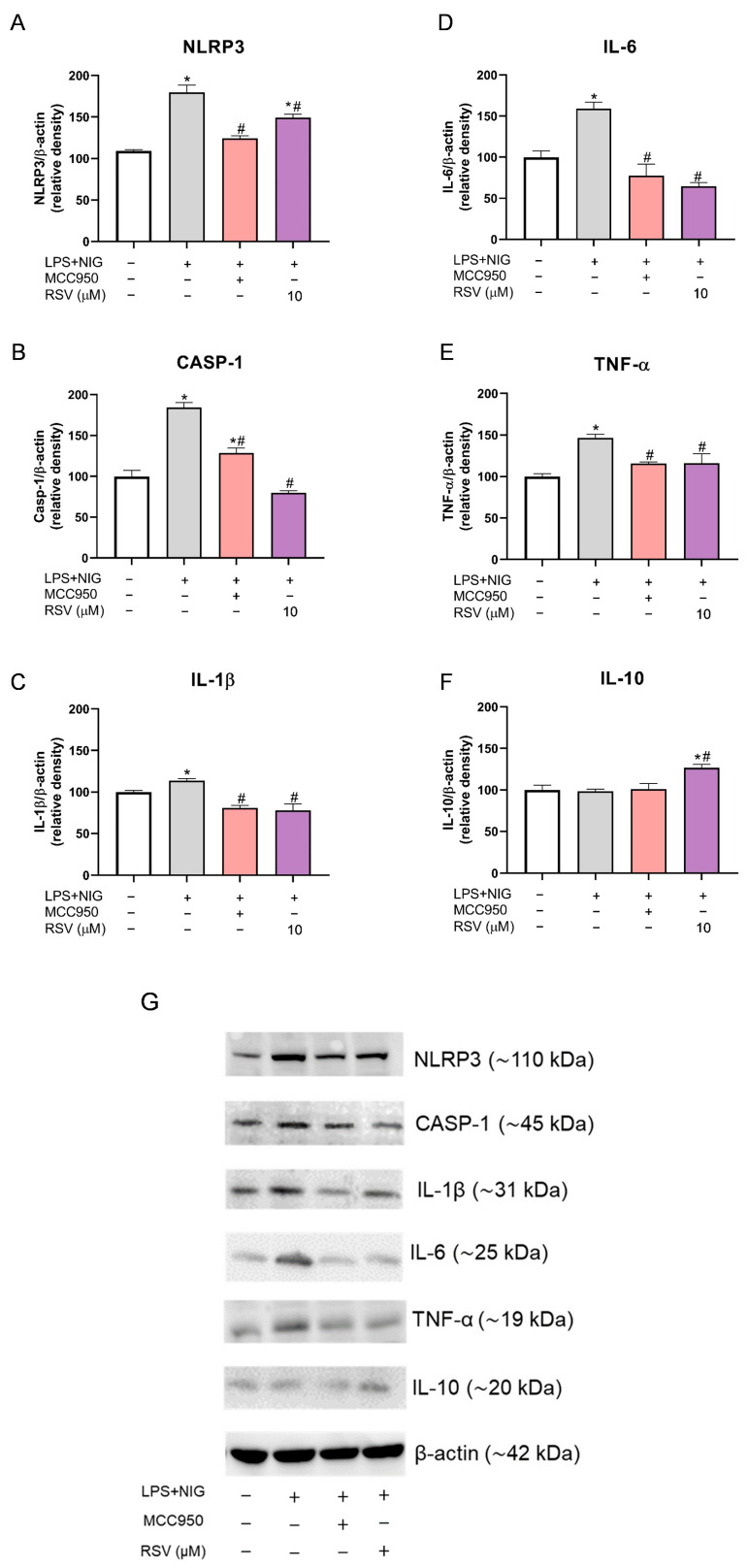
Western blot analysis shows RSV modulation in the relative protein density of inflammatory markers. (**A**) Relative protein density of NLRP3; (**B**) relative protein density of caspase-1; (**C**) relative protein density of IL-1β; (**D**) relative protein density of IL-6; (**E**) relative protein density of TNF-α; (**F**) relative protein density of IL-10; (**G**) Western blot analysis showing the respective protein bands. The experiments were performed in triplicate. The statistical analysis was conducted using one-way ANOVA followed by the Tukey post hoc test. *p* < 0.05 was considered significant. Data are expressed as mean values ± SEM. * means comparison to the control group; # means comparisons to the activation group (LPS + NIG).

The mRNA levels and relative protein density of NLRP3, CASP-1, IL-1β, IL-6, TNF-α, and IL-10 in microglial cells exposed to NLRP3 inflammasome activation conditions and pre-treated with RSV prior to NLRP3 inflammasome oligomerization were determined in this study (Figure 6 and Figure 7).

All mRNA levels of the pro-inflammatory gene increased in cells exposed to LPS + NIG (activation control), compared to the negative control (*p* < 0.0001) (Figure 6A–E). On the other hand, for IL-10, no significant increase was observed (Figure 6F). The MCC950 inhibitor was potent in decreasing all pro-inflammatory mRNAs (Figure 6C–E), NLRP3, and CASP-1 (Figure 6A,B) when compared to the activation control (LPS + NIG) (*p* < 0.0001). An increase in IL-10 was detected compared to the control (*p* = 0.0011) and the activation control (*p* = 0.0413) (Figure 6F).

When added to the cells as a pre-treatment, RSV showed overexpressed pro-inflammatory mRNA levels compared to the control (Figure 6A–E). Nevertheless, RSV was satisfactory in reducing inflammasome components (NLRP3 and CASP-1) when compared to the activation group (LPS + NIG) using 1 μM (*p* < 0.0001), 10 μM (*p* < 0.0001), and 25 μM (*p* < 0.0001; *p* = 0.0151) (Figure 6A,B). In addition, a decrease in the IL-1β (1 µM—*p* = 0.0004; 10 μM—*p* = 0.0062; 25 µM—*p* = 0.0009), IL-6 (1 µM—*p* < 0.0001; 10 μM—*p* = 0.0001; 25 µM—*p* = 0.0022), and TNF-α (1 µM—*p* = 0.033; 10 μM—*p* = 0.0048; 25 µM—*p* < 0.0001) mRNA levels was evident when compared to the activation control (LPS + NIG) (Figure 6C–E).

Although the mRNA levels for IL-10 did not present statistically significant results in the activation control, the MCC inhibitor (*p* = 0.0413) and 1 and 10 μM of RSV (*p* < 0.0001) increased this measure, presenting more significance than MCC950; 1 μM and 10 μM of RSV elevated the IL-10 mRNA levels compared to the activation control (Figure 6F).

The intermediate concentration of RSV (10 μM) was tested for Western blot analysis to perform protein density quantification (Figure 7), and the respective protein bands are shown in Figure 7G (entire membranes are given in Supplementary Material S2). The density of NLRP3 and CASP-1 was increased in microglia exposed to inflammasome activation conditions (*p* = 0.0008; *p* < 0.0001); however, MCC (*p* = 0.0025; *p* = 0.0006) and RSV (*p* = 0.0231; *p* < 0.0001) were effective in reducing these proteins (Figure 7A,B).

Pro-inflammatory cytokines (IL-1β, IL-6, and TNF-α) increased in cells with activated inflammasomes (Figure 7C–E), in agreement with the mRNA level results (*p* = 0.048; *p* = 0.009; *p* = 0.0023). MCC and 10 μM of RSV restored the protein levels of these markers, showing statistical significance only concerning the activation control (LPS + NIG) (Figure 7C–E). For IL-1β, the *p* values were 0.0201 and 0.0147, respectively. For IL-6, *p* = 0.0012 for MCC and *p* = 0.0004 for 10 μM RSV. The TNF-α *p* values were 0.0298 and 0.0316, respectively.

Again, although IL-10 did not show any difference between the control and activation control, 10 μM of RSV was effective in increasing the levels of this anti-inflammatory cytokine (*p* = 0.0347 compared to the control and *p* = 0.0262 compared to the activation control) (Figure 7F).

## 3. Discussion

RSV is a polyphenol found in more than 70 plant species, such as peanuts and grapes [30,32]. Many studies have already demonstrated that RSV has medicinal effects related to the ability to modulate several inflammatory pathways, including the inflammasome [34,35,36,37,40]. However, there is no proof obtained through a methodology that uses priming and assembly signals of NLRP3 in microglia. Therefore, this research adopted an innovative method to prove the inhibitory action of RSV on the NLRP3 inflammasome in association with the P2X7 and A1 purinergic receptors for the first time.

Initially, we evaluated, through in silico methods, the existence of ligation between RSV and the PYD region of the NLRP3 inflammasome, as this is the same domain where MCC950, a well-known NLRP3 inhibitory molecule, is capable of interacting to block its oligomerization [41,42]. Thus, it was also possible to compare the inhibitory potential of a synthetic molecule and one obtained from products of natural origin.

The affinity values between the target and ligand indicate biological compatibility between RSV and NLRP3 inflammasome PYD. However, the most promising result was presented by MCC950, indicating higher interaction potential than RSV. However, since MCC950 is a complete NLRP3 inhibitor, the results obtained for RSV could indicate a positive aspect as a partial inhibitor, perhaps bringing the NLRP3 inflammasome to basal levels under inflammatory situations. The RMSD measures the distance between the ligand atoms and the target, with values less than 2 Å indicating the quality of the methodological process [43,44]. From this metric, it can be seen that RSV and MCC950 are compatible with the PYD domain.

The results for Ki reflect the ligand concentration necessary to inhibit half of the target’s maximum activity; the lower this value, the greater the inhibitory potential of the molecule. Despite the lower value for MCC950 (696.25 nM), it is highlighted that this result is an estimate calculated from the three-dimensional conformations of the target and ligand molecules and their binding energies. These values cannot correspond to those found in biological experimental models [43,44].

Although the molecular docking results are better for MCC950, it can be proven that RSV has a favorable inhibitory affinity, with a negative value of 6.7 and 12.27 µM necessary to inhibit half of the NLRP3 activity. MCC950, as a synthetic compound, can cause adverse effects when used as a treatment and act as a total inhibitor of NLRP3 [27]. Natural agents can be partial modulators, reducing intense inflammatory activations but without inhibiting what would be basal and consequently presenting fewer adverse effects [27,45,46]. Thus, this is a promising natural compound that can inhibit the NLRP3 complex by binding in the PYD domain.

Despite the above, our in silico results also demonstrate that RSV has a simple molecular structure and exhibits exceptional affinity for the PYD domain of NLRP3, with binding free energy values (ΔG = −22.44 ± 5.91 kcal/mol) that are significantly more favorable than those observed for the reference inhibitor MCC950 (−13.63 ± 11.08 kcal/mol). These findings not only corroborate previous reports on the ability of RSV to modulate inflammatory pathways through multiple mechanisms [31,33] but also validate our experimental data, which demonstrated its effectiveness in reducing NLRP3, caspase-1, and IL-1β expression in activated microglia (discussed below).

Molecular dynamics analysis revealed that the RSV–PYD complex exhibited lower structural fluctuations (RMSD = 2.17 ± 0.33 Å) compared to both the APO system (2.26 ± 0.33 Å) and MCC950 (3.25 ± 0.60 Å), indicating the remarkable stabilization of the binding site. This pattern was confirmed by the reduced fluctuation in key residues (RMSF) and greater overall structural compactness (Rg = 21.55 ± 1.22 Å), suggesting that RSV effectively restricts PYD domain mobility. This effect may explain its unique ability to inhibit inflammasome oligomerization in vitro without causing the complete suppression characteristic of irreversible inhibitors such as MCC950.

The per-residue energy decomposition analysis highlighted fundamental differences in binding modes: while RSV showed stronger interaction with Leu39 (ΔG = −3.57 kcal/mol), MCC950 preferentially bound to Ile37 (ΔG = −2.40 kcal/mol). This distinction reflects the fact that different pharmacological profiles of RSV promote more balanced modulation by stabilizing regions critical for PYD oligomerization, whereas MCC950 acts more drastically. These theoretical findings correlate well with our experimental results, in which RSV preserved microglial homeostasis by simultaneously reducing P2X7 expression and the ROS levels while increasing IL-10 and A1 receptor expression, for example.

Our observations corroborate findings that demonstrate the ability of flavonoids derived from *Euterpe oleracea* (apigenin, catechin, and epicatechin) to modulate NLRP3 activation [47,48]. Although RSV was not evaluated in these studies, our data indicate that it shares similar structural and dynamic properties with these phenolic compounds, interacting with the same PYD domain and promoting conformational stabilization compatible with functional inhibition. This convergence of mechanisms reinforces the therapeutic potential of natural NLRP3 modulators, which combine anti-inflammatory efficacy with cellular homeostasis preservation, as evidenced by the absence of cytotoxicity and positive modulation of anti-inflammatory pathways.

Remarkably, RSV surpassed the flavonoids in terms of binding affinity (ΔG = −22.44 kcal/mol vs. −7.1 to −21.14 kcal/mol), suggesting potential advantages in potency and interaction stability. These findings position RSV as an outstanding therapeutic candidate for conditions driven by NLRP3 hyperactivation, offering a unique balance between efficacy and safety. Thus, RSV emerges as a natural NLRP3 modulator with dual potential to inhibit exacerbated inflammation through the conformational stabilization of the PYD domain while simultaneously preserving cellular homeostasis by modulating anti-inflammatory pathways—a distinctive pharmacological profile that clearly differentiates it from synthetic inhibitors such as MCC950.

Therefore, aiming to use RSV as a treatment for neuroinflammatory diseases through the modulation of the NLRP3 inflammasome, we prioritized analyzing this molecule isolated in microglial cells of the murine BV-2 cell line in the second stage. Through basic tests, we verified possible changes in cellular homeostasis that RSV could cause by itself. In this context, it is important to note that we used a specific positive damage control for each test (MTT and PicoGreen—dimethylsulfoxide (DMSO) reagent; nitrite levels—sodium nitroprusside (SNP) reagent; ROS levels—H_2_O_2_ reagent), which guaranteed the functionality of the test and that the results presented were reliable. Unusual behavior of the DMSO and H_2_O_2_ controls for dsDNA, as well as the ROS levels, was observed during the time periods analyzed. It is important to mention that each incubation time had a positive control due to the capacity of the cells (in general) to try to recover their homeostasis, even under stress conditions. This capacity for recovery could explain why we observed a difference between the incubation periods for dsDNA and ROS release in the control groups, which is not conducive to comparing the results of these controls at different times (24 versus 48 versus 72 h). Furthermore, the DMSO and H_2_O_2_ results (raw data) were normalized to those of the negative control group for each corresponding incubation time. Despite this, using these positive controls, we found a statistically significant increase in dsDNA and ROS release for all incubations, proving that the assay performed reflected the real results. Therefore, we considered the RSV concentration curve (0.01–100 μM) at three exposure times (24, 48, and 72 h).

Few metabolic cell changes are observed at intermediate RSV concentrations (0.1–25 μM) in BV-2 cells. Only the two highest (50 and 100 µM) deserve attention and indicate that caution is needed for their use, since more significant changes that are harmful to cells could be seen, especially when *p* < 0.001, as demonstrated. Furthermore, for these concentrations, a cell viability reduction is evident for all analyzed times. Interestingly, the lowest concentration of RSV (0.01 μM) decreased the cell viability at all times analyzed. Concentration-dependent effects are not necessarily expected when using natural product molecules (as observed here). This effect could be explained by the hormetic pharmacology phenomenon, which is found in many products when applying a curve composed of many concentrations, including RSV [49,50,51]. In addition, when RSV was in contact with cells for 72 h, cells that were still alive showed an increased proliferation rate compared to previous incubation times (24 and 48 h). This effect could occur due to RSV’s beneficial properties, perhaps providing a suitable environment for cellular growth based on its bioactive properties, as well as its antioxidant capacity, for example [32,33,52].

According to the results, there was considerable membrane damage with the exposure of free dsDNA in the supernatant, indicating cell disruption/death and harmful changes to cellular metabolism and homeostasis. The dsDNA release levels are not dependent on the time of exposure to RSV. We observed that the statistical changes were more significant in the first time period—24 h. The nuclease enzymes present in the medium or secreted by the cells degrade DNA, which would explain the significant decrease in dsDNA levels at 48 and 72 h. Another explanation for our results is the antioxidant and pro-survival effects. For example, after an initial cell death response and DNA release (especially at higher concentrations), surviving cells may activate autophagy or repair mechanisms (through RSV’s action), reducing the continued release of extracellular DNA.

According to these explanations, it is suggested that the safest concentrations of RSV to be used in vitro and to treat inflammatory diseases are between 0.1 and 25 µM. The results presented here are the concentrations used in scientific references that have applied RSV in models of diseases that affect the CNS and present inflammatory characteristics, ranging from 5 to 25 µM [53,54,55].

Recent research found that RSV is safe in concentrations lower than 25 μM in RAW 264.7 cells, and, from 50 μM, the cell viability decreases by 64.42% [56]. Additionally, a review study indicates that RSV’s cytotoxic effects are directly affected by cellular mitochondria, per our findings, as measured via the succinate dehydrogenase viability in the MTT test [57]. Concentrations above 50 µM are cytotoxic as they impair cellular energy homeostasis, affecting respiration, the mitochondrial structure, and the membrane potential. They activate the cell death pathway through apoptosis, with changes in calcium homeostasis, the activation of caspases, and the release of cytochrome c [57,58,59]. However, at low concentrations (<50 µM), RSV can activate cytoprotective mechanisms by increasing antioxidant protection and reducing ROS and NO, including at the mitochondrial level [57,60].

After confirming the in silico interaction of RSV with the NLRP3 PYD domain and studying the behavior of microglia exposed to this molecule, we continued to evaluate it in an innovative model of NLRP3 inflammasome activation in the BV-2 cell line of microglia. Our results, for the first time, reveal the preventive mechanism of action of RSV in this pathway through the P2X7 receptor, avoiding NLRP3 oligomerization.

Cells exposed to the inflammasome activation conditions (LPS + NIG) demonstrated impaired cell viability, along with an increase in nitrite and ROS levels. NLRP3 inflammasome activation decreases the mitochondrial activity of the enzyme succinate dehydrogenase, leading to mitochondrial dysfunction and the release of ROS and reactive nitrogen species, such as NO. This mechanism could be explained by the passage of electrons, which should transit between the mitochondrial complexes for ATP formation but now pass through the pore openings of the mitochondrial membrane, leading to the greater production of ROS and NO [61,62,63]. Inverse results were observed for MCC950.

Regarding RSV, mainly at the 25 µM concentration, it recovered cell viability; this effect was statistically similar to that in the control group but different from that in the activation control. Surprisingly, all concentrations of RSV reduced the levels of nitrite and ROS compared to the activation control. Still, the 5 and 10 µM concentrations for nitrite and 1 µM for ROS stood out, as some concentrations did not show a statistical difference from the negative control but only from the activation control. RSV reduces ROS production and nitrite levels through multiple interconnected mechanisms [30,52]. It acts as a direct antioxidant by scavenging ROS such as superoxide anion and peroxynitrite and inhibits the activity of NADPH oxidase, a primary source of ROS in inflammatory cells [30,60]. It activates the Nrf2 pathway, promoting the expression of antioxidant enzymes (superoxide dismutase, catalase, and glutathione peroxidase, for example), and modulates nitric oxide synthase activity, inhibiting iNOS under inflammatory conditions, thereby reducing excessive NO production and subsequent nitrite levels [34]. Additionally, it improves mitochondrial function via the activation of SIRT1 and exerts anti-inflammatory effects by inhibiting the NF-κB and MAPK pathways, contributing to a cellular environment with lower oxidative and nitrosative stress [57,64]. Bartra et al. [55] confirmed the protective effects of RSV via the modulation of the SIRT1, Nrf2, and NF-κB pathways in order to reduce inflammation and induce antioxidant enzymes. Furthermore, they propose that SIRT1 mediates the antioxidant and anti-inflammatory mechanisms in LPS-activated microglial cells [55].

Therefore, 1, 10, and 25 µM were chosen for the cytometer and RT-qPCR analyses and 10 μM for protein density measurement (an intermediate concentration of RSV used in the curve, which had an effect in the initial experiments).

The cell cycle results may confirm the microglial activation, with a significant increase in cells in the S phase under LPS + NIG exposure, characterizing the cell cycle arrest in this phase, induced by pro-inflammatory factors [65]. The increase in cells at the S phase suggests that these cells do not complete the cell cycle and achieve the G2/M phases. These results could explain why we observed a decrease in cellular viability/number in the MTT assay for the LPS + NIG group. Microglia, when active, recruit immune system components, including NLRP3 inflammasome activation [65,66,67]. Meanwhile, MCC950 again showed the opposite results, remaining in the G0/G1 phase of cell rest, blocking replication, and reducing intense cell proliferation and possible uncontrolled inflammation. All RSV concentrations (1, 10, and 25 µM) increased the G0/G1 phase, compared to the cell and activation controls, indicating the existence of microglia in a resting period or in preparation for division. However, in the S phase, only the activation control increased this parameter, while RSV had the opposite effect compared to inflamed cells. It can be assumed that RSV can slow down or even block the activated cell’s replication, remaining at rest for longer. Only a decrease in the G2/M phase was observed, with no difference between the activation or inhibition controls and RSV pre-treatments. It is believed that, due to the short protocol time (6 h), it was impossible to visualize the complete replication cycle of microglia (12–24 h) without changes in this G2/M phase.

Depending on the cell type and pathophysiological context, RSV can affect the cell cycle through different mechanisms. For example, in hepatocellular carcinoma cells, it can induce G2/M phase arrest [68]. In mononucleated cells from rat brains induced in a model of multiple sclerosis, RSV exhibited similar actions to those found in our research, with a decrease in the S phase and an increase in the cell rest phase [69]. Furthermore, in another study, 25 µM of RSV decreased cell proliferation, verified by analyzing the cycle of astrocytes and microglia in an in vitro Alzheimer’s disease model, suggesting that inhibition of the NF-κB pathway is responsible for preventing cell cycle progression in inflammatory conditions [70].

The purinergic receptor P2X7 is activated during inflammation, while the A1 receptor activates neuroprotective/anti-inflammatory mechanisms [11,12,13,71]. The analysis of these receptors in cells with an activated NLRP3 inflammasome is essential to verify the modulation of inflammatory processes and evaluate the inflammatory balance and bioactive properties of RSV in this context, which has not yet been reported.

The P2X7 receptor (activated by extracellular ATP) is essential in releasing inflammatory cytokines, such as IL-1β and IL-18, and activating the NLRP3 inflammasome [11]. The A1 receptor acts as an anti-inflammatory mediator, activated by adenosine (an ATP metabolite) to negatively regulate inflammation [13]. For example, it inhibits the production of pro-inflammatory cytokines, modulates the activity of immune cells, and promotes tissue protection in situations of cellular injury or stress [71]. Therefore, while P2X7 promotes inflammation, A1 acts as an anti-inflammatory counterbalance. The interaction between these receptors reflects the dynamic regulation of the inflammatory process. Dysregulation of ATP and adenosine signaling is implicated in several chronic inflammatory diseases [72]. Examining the interaction between these receptors can reveal potential points of intervention.

Priming and assembly signals increase the P2X7 expression and protein levels, developing pro-inflammatory factors. P2X7 receptor activation can induce and activate the NLRP3 inflammasome [16,73,74,75]. This process can be interconnected and crucial in regulating the inflammatory and immune response. It is essential for host defense, but, in excess, it is also harmful [11,12,76].

The results reveal that the three RSV concentrations and MCC950 restored the expression values of this receptor compared to the control and activation control. Therefore, it is suggested that RSV modulates the P2X7 receptor, controls neuroinflammation, and reduces tissue damage generated by intense receptor and inflammasome activation.

Consequently, it can prevent the release of pro-inflammatory factors and regulate the activation of the NLRP3 inflammasome, which, in an exacerbated manner, would cause tissue damage [76,77]. At present, our results are the first to demonstrate the joint action of RSV on the P2X7 receptor and the NLRP3 inflammasome.

For the A1 purinergic receptor, a loss of neuroprotective effects was observed through a decrease in its expression and protein levels during NLRP3 activation, but, again, RSV managed to reverse this effect, increasing A1 receptor expression and the protein density. Among the mechanisms through which it exerts neuroprotective effects, the inhibitory capacity for the release of excitatory neurotransmitters, such as glutamate, stands out, reducing neuronal excitotoxicity and attenuating the CNS inflammatory response by reducing the production of pro-inflammatory cytokines—mainly TNF-α and IL-1β—and inhibiting the activation of immune cells, such as microglia [13,19].

In a study with an animal model for intracerebral hemorrhage, it was seen that the neuroprotective action of RSV was linked to the adenosine A1 receptor. This activation resulted in an improvement in behavioral parameters in rats and also in the morphological tissue characteristics of the brain and a decrease in the expression of TNF-α [78].

Some studies suggest that RSV exerts effects in terms of controlling ATP and ADP degradation through the modulation of enzymes and purinergic receptors [38,79,80,81]. For this, we hypothesize that the stimulation of microglia with LPS and NIG promotes cell activation and ATP release as a danger signal that binds to the P2X7 receptor. RSV controls ATP degradation and release, explaining how this treatment regulates the receptor (less expression) and reduces the NLRP3 mRNA levels and protein density. However, the A1 receptor’s overexpression by RSV could be explained by adenosine-enhancing agents inhibiting degradation and elevating the endogenous adenosine levels to control the protective responses in the brain by potentiating A1 receptor activation [13,80].

The P2X7 receptor and the NLRP3 inflammasome in microglia proliferate (cell cycle), inducing the production and release of pro-inflammatory cytokines, such as IL-6, IL-1β, and TNF-α, to eliminate the stressor [65]. Upon analyzing the inflammatory mRNA and protein levels, we can also confirm the direct action of RSV in modulating these cytokines.

Exposure to LPS + NIG increased the cytokine levels, which was reversed with the use of RSV, attenuating the exacerbated inflammation. On the other hand, IL-10 is an anti-inflammatory cytokine responsible for restoring cellular homeostasis in pro-inflammatory conditions [82]. It was evident that 1 and 10 µM of RSV, when compared to the action of MCC950, acted as a neuroprotector, with better results in increasing IL-10, inhibiting microglial activation and inflammation.

Furthermore, the NLRP3 and CASP-1 mRNA and protein levels (inflammasome components) were decreased with RSV pre-treatment. It is possible to note that ROS levels are also reduced with RSV, which contributed to helping to restore the expression of these inflammasome components, as, when increased, they function as inducers for the formation and activation of NLRP3 [3,62]. Recent studies highlight the P2X7/NLRP3/IL-1β pathway in neurological diseases [18,83,84,85,86,87]. In a mouse epilepsy protocol, the gene expression of P2X7, NLRP3, and IL-1β was increased [83]. Moreover, in an animal model with a depression-like phenotype, the P2X7/NLRP3/IL-1β pathway was noted as a potential mechanism for the study of treatments that target this pathological condition [87].

RSV exhibits pleiotropic properties, and certain effects may only occur at specific concentrations due to the modulation of multiple molecular pathways in a concentration-dependent manner [30,50]. Evidence from the literature indicates that lower concentrations of RSV (<50 µM) tend to activate cytoprotective and antioxidant pathways, such as SIRT1. In contrast, higher concentrations (>50 µM) induce alterations in calcium homeostasis, mitochondrial membrane potential disruption, and caspase activation, ultimately leading to apoptosis—particularly in cancer cells [57,58]. In this context, Calabrese et al. [50] also emphasize that high RSV concentrations are required to trigger pro-apoptotic mechanisms in tumor cells, while concentrations below 50 µM are more associated with antioxidant and anti-inflammatory effects [50]. Therefore, the cellular responses to RSV appear to be concentration-dependent, which may explain the variability observed across different studies and underscores the importance of considering the concentration as a key factor when interpreting experimental outcomes.

In summary, this is the first study that shows the anti-inflammatory potential of RSV through the modulation of the NLRP3 inflammasome in association with purinergic receptors, such as P2X7 and A1. This characterizes this scientific investigation as an innovative approach, bringing new perspectives that will allow for future investigations.

## 4. Materials and Methods

### 4.1. Computer Simulation

#### 4.1.1. Molecular Docking

Molecular docking simulations were performed to investigate the interaction between RSV and the NLRP3 protein PYD region (pyrin domain). The AutoDock Vina software version 1.1.2 (AD Vina) integrated with the AMDOCK tool was used [43,88]. The target was the PYD domain of NLRP3, the same area where MCC950 inhibits the inflammasome [27]. The chemical structure of the PYD region was obtained from the Protein Data Bank (PDB ID: 2NAQ), and the ligand molecules, RSV (ID: 445144) and MCC950 (ID: 9910393), were obtained from the PubChem database. The complexes formed were evaluated and analyzed regarding their binding energy/affinity, inhibition constant (estimated Ki), and RMSD (distance between atoms). The Functional Transformation SITE (FTSITE) tool was used to identify and visualize binding sites between ligand targets [88]. Similar computational methodologies have been employed in previous studies by our research group [47,89,90]. The molecular docking process was carried out using the AMDOCK software 1.6.2 version, and Pymol 3.1 version (Schrödinger, LLC, New York, NY, USA) was used for visualization and image generation.

#### 4.1.2. Molecular Dynamics Simulation

The three-dimensional structures of the ligands were optimized at the HF/6-31G* level using the Gaussian09 software [91], and partial atomic charges were calculated using the RESP method [92]. System preparation was performed using the tLEaP module from the AMBER package [93], applying the GAFF force field for the ligands and ff14SB for the protein [94]. The PYD domain (PDB ID: 2NAQ) was protonated at physiological pH (7.4) using the H++ server [95] and solvated in a TIP3P water box [96,97] with periodic boundaries. Ions were added to neutralize the system and simulate an ionic strength of 0.15 M, reproducing physiological conditions.

The systems were subjected to a multi-step minimization protocol involving solvent relaxation, the relaxation of protein hydrogens, the joint relaxation of protein and solvent hydrogens, and global system minimization. The hybrid steepest descent/conjugate gradient algorithm was used at all stages to ensure efficient energy convergence. This protocol was developed based on methodologies previously established in our studies [48,89,98]. The simulations were conducted for 100 ns in the isothermal–isobaric ensemble (NPT), maintaining a constant temperature and pressure using a Langevin thermostat [99] and Berendsen barostat [100]. The trajectories generated were used for structural analyses and binding free energy calculations.

#### 4.1.3. Generalized Born and Surface Area Continuum Solvation Calculations (MM/GBSA)

The affinity between the ligands and NLRP3 was quantified using the MM/GBSA method [101], available in the AmberTools23 package [93]. The calculations were based on 500 snapshots extracted from the last 10 ns of the molecular dynamics simulations. The energy components considered included van der Waals interactions, electrostatic contributions, polar solvation (generalized Born model), and non-polar solvation (solvent-accessible surface area). Energy decomposition per residue was carried out to identify the most relevant residues contributing to the stabilization of the complexes. The mathematical foundation underlying the binding free energy calculations has already been previously published [102].

### 4.2. Microglia Cell Culture

A microglia cell line (BV-2) (Rio de Janeiro Cell Bank Code—0356, Rio de Janeiro, RJ, Brazil) was cultured with RPMI-1640 medium (Vitrocell*^®^*, Campinas, SP, Brazil) modified to contain 10 mM HEPES (Ludwig*^®^*, Alvorada, RS, Brazil) with 10% fetal bovine serum (FBS) (Vitrocell*^®^*, Campinas, SP, Brazil) and supplemented with 1% of antibiotics (100 U/mL penicillin; 100 mg/mL streptomycin) (Vitrocell*^®^*, Campinas, SP, Brazil). Cells were cultured at 37 °C with 5% CO_2_ and atmospheric oxygen.

### 4.3. Resveratrol In Vitro Safety Profile

**Figure 8:**
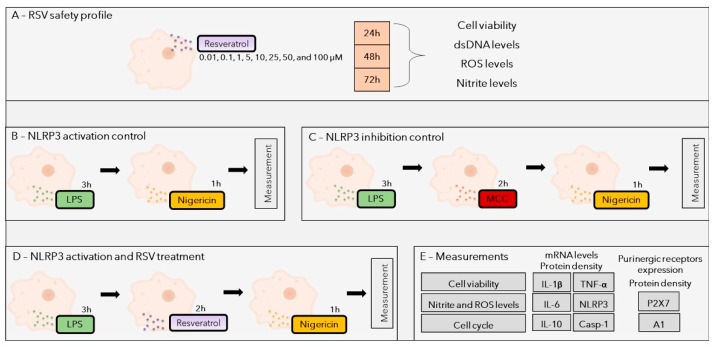
An illustrated scheme of the in vitro experiments performed in this study. (**A**) Investigation of RSV cytotoxicity in BV-2 cells. Microglial cells were exposed to a concentration curve of RSV for 24, 48, and 72 h, followed by cell viability, dsDNA, ROS, and nitrite level measurements; (**B**) scheme of exposure to treatments that induced NLRP3 activation; (**C**) scheme of exposure scheme to treatments that inhibited NLRP3 activation for the inhibition control; (**D**) scheme of exposure to RSV treatment to test its action in the NLRP3 inflammasome; (**E**) list of measurements performed in (**B**–**D**) treatments.

RSV was tested in BV-2 cells for its safety profile. RSV (C14H12O3; molecular weight 228, 25 g/mol; Sigma-Aldrich*^®^*, St. Louis, MO, USA) was initially diluted in DMSO (Sigma-Aldrich*^®^*, St. Louis, MO, USA) and RPMI-1640 medium (1% DMSO in initial solution), followed by dilutions into the culture medium, and added to the cells at concentrations of 0.01, 0.1, 1, 5, 10, 25, 50, and 100 µM [103]. In RSV 100 µM, the existing percentage of DMSO was 0.1% (non-toxic to BV-2 cells). The incubation times were 24, 48, and 72 h [47,104,105]. After incubation, the cell viability, extracellular double-strand DNA quantity (dsDNA), nitrite (indirect nitric oxide—NO measurement), and ROS levels were evaluated (Figure 8A). In this step, controls to induce cell damage were used: 15% DMSO for cell viability and dsDNA, 10 µM SNP (Sigma-Aldrich*^®^*, St. Louis, MO, USA) for nitrite levels, and 25 µM hydrogen peroxide (H_2_O_2_, Sigma-Aldrich*^®^*, St. Louis, MO, USA) for ROS levels [47,104,105,106,107]. Moreover, the negative damage control (control) group was composed of cells without treatment. It is important to mention that this experiment was conducted in triplicate and with the corresponding assays.

### 4.4. Resveratrol Pre-Treatment and NLRP3 Inflammasome Activation

Initially, 1 µg/mL of LPS (Sigma-Aldrich*^®^*, St. Louis, MO, USA) was added to the microglia cells for 3 h [20,108]. After priming–inducing factor addition, the same cells were treated with RSV (0.01–100 µM) for 2 h [20,103,109], followed by 10 µM NIG (Sigma-Aldrich^®^, St. Louis, MO, USA) exposure for 1 h [47,109]. RSV was used to test its anti-inflammatory capacity through the prevention of NLRP3 inflammasome oligomerization, potentially acting as an inhibitor between the use of LPS (increases the inflammasome transcription factors) and NIG (induces the assembly (oligomerization) of the inflammasome proteins). As an inhibition control, 100 nM of MCC950 (Sigma-Aldrich*^®^*, St. Louis, MO, USA) was added, between the use of LPS and NIG, for 2 h [20,109]. MCC950 is already known to inhibit the oligomerization of the NLRP3 inflammasome’s ASC domain. We also used an activation control; for this, LPS and NIG were added to the cells at the exact times described here. This innovative in vitro protocol for the activation of the NLRP3 inflammasome followed guidelines from Zhou et al. [20], Davidson et al. [47], and El Sabbagh et al. [109] with modifications. The experimental model is schematized in Figure 8B–E. After all incubations, the cell viability, parameters related to oxidative stress, cell cycle arrest, and the expression of purinergic receptors, cytokines, CASP-1, and NLRP3 were evaluated. This experiment was conducted in triplicate, as well as the corresponding assays.

### 4.5. Experimental Assays

#### 4.5.1. Determination of Cell Viability and Extracellular Double-Strand DNA

Cell viability was evaluated using the MTT assay. After the removal of the supernatant, the cells were incubated with 20 μL of MTT reagent (3-(4,5-dimethyl-2-thiazolyl)-2,5-diphenyl-2H-tetrazolium bromide) (Sigma-Aldrich^®^, St. Louis, MO, USA) at 5 mg/mL and 200 μL of PBS 1X for 1 h at 37 °C. After incubation, the reagent was removed and 200 μL of DMSO was added to solubilize the formazan crystals generated by the mitochondrial succinate dehydrogenase enzyme. Absorbance was determined at 560 nm in the plate reader SpectraMax I3 (Molecular Devices, San Jose, CA, USA) [110].

dsDNA was quantified in the cellular supernatant using 96-well black plates. First, 10 μL of PicoGreen^®^ dsDNA reagent (Thermo Fisher Scientific^®^, Waltham, MA, USA), diluted 1:200 in Tris–EDTA 1X buffer, was added to 10 μL of the samples and 80 μL of Tris-EDTA 1X buffer for 5 min at room temperature, avoiding light exposure. The fluorescence intensity was determined at 480 nm of excitation and 520 nm of emission (SpectraMax I3, Molecular Devices, San Jose, CA, USA) [111].

#### 4.5.2. Nitrite and ROS Level Measurement Analysis

Using an indirect protocol to detect NO, organic nitrite was measured. First, 50 μL of Griess reagent (sulfanilamide 1% and N-1-naphthylenediamine-bihydrochloride 0.1%, proportion—1:1) was added to 50 μL of the supernatant sample for 15 min at room temperature and under light protection. The absorbance was determined at 540 nm (SpectraMax I3, Molecular Devices, San Jose, CA, USA) [112].

To measure ROS production, 0.1 mM of 2′-7′ dichlorodihydrofluorescein diacetate (DCFH-DA) reagent (10 μL) (Sigma-Aldrich*^®^*, St. Louis, MO, USA) was added to 50 μL of the sample and 100 mM of Tris–HCl buffer (65 μL). This assay was carried out using black 96-well plates. A fluorescence reading was performed at excitation of 485 nm and emission of 520 nm after 1 h of incubation at room temperature with light protection (SpectraMax I3, Molecular Devices, San Jose, CA, USA) [113].

#### 4.5.3. Cell Cycle Evaluation

With the three most effective concentrations of RSV determined through the previous assays, the treatments were performed again to carry out the cell cycle evaluation. After the treatment protocols, propidium iodide (PI) (Sigma-Aldrich*^®^*, St. Louis, MO, USA) was used in this analysis. Cells were collected and washed with PBS 1X to remove the medium and other debris. After washing, microglia were centrifuged at 1500 rpm for 5 min (low-speed differential centrifugation). The pellet cells were fixed with 70% ice-cold ethanol and centrifuged again for 5 min at 1500 rpm. The cell pellets were resuspended in a solution containing 0.05% Triton, 100 µg/mL RNase, and 50 µg/mL PI, following 30 min of incubation at 37 °C. The BV-2 microglial cells were analyzed using a flow cytometer (BD FACSVerse; BD Biosciences, San Jose, CA, USA), and the data were examined using the FlowJo V10 software (FlowJo, Ashland, OR, USA) [114].

#### 4.5.4. Flow Cytometer Analysis

The three most effective concentrations of RSV were determined previously via the initial assays to perform the analysis of A1 and P2X7 receptor expression using a flow cytometer (BD FACSVerse; BD Biosciences, San Jose, CA, USA), and the quantification was performed using the FlowJo V10 software (FlowJo, Ashland, OR, USA). BV-2 microglial cells were fixed in 4% paraformaldehyde (PFA) and washed with PBS containing 2% FBS. Primary antibodies for A1 and P2X7 purinergic receptors (1:500, Life Technologies, Carlsbad, CA, USA) were used in the samples for 35 min. After washing with PBS, Alexa Fluor 555 or 647 secondary antibodies (1:500, Life Technologies, Carlsbad, CA, USA) were added for the same incubation time. In the flow cytometer, forty thousand events were acquired for the samples. Forward and side light-scatter signals were used to exclude dead cells and debris [115].

#### 4.5.5. RT-qPCR Analysis

RNA isolation followed the manufacturer’s standards, using Trizol reagent (TRI Reagent—Sigma-Aldrich*^®^*, St. Louis, MO, USA). Samples were quantified (RNA ng/μL) using a NanoDrop Lite (Thermo Fisher Scientific*^®^*, Waltham, MA, USA) and cDNA conversion using a commercial iScritTM cDNA synthesis kit (Bio-Rad, CA, USA). IL-1β, IL-6, IL-10, TNF-α, NLRP3, and CASP-1 mRNA quantification was conducted using specific primers (Supplementary Material S3) (Ludwig*^®^*, Alvorada, RS, Brazil) and the components provided in the commercial GoTaq*^®^* qPCR master kit (Promega, Fitchburg, WI, USA) in a QuantStudio 5 real-time PCR device (ThermoFischer Scientific, Waltham, MA, USA). The cycle conditions were as follows: 50 °C—2 min, 95 °C—2 min and 40 cycles of 95 °C—15 s and 60 °C—30 s. The mRNA level data were analyzed using delta–delta Ct calculation and relative expression conversion. It is important to mention that these analyses were carried out using the three main concentrations of RSV that presented effective potential in the initial experiments.

#### 4.5.6. Western Blot Analysis

Finally, one concentration of RSV was selected to assess the protein expression of different important proteins involved in the inflammatory cascade and purinergic system. This selection was based on the intermediate concentration of RSV used in the curve. Microglia were homogenized in ice-cold radioimmunoprecipitation assay buffer with 1 mM protease and phosphatase inhibitors. The protein concentrations were determined using the Lowry methodology (Sigma-Aldrich). Samples were separated using sodium dodecyl sulfate–polyacrylamide gel electrophoresis and transferred into nitrocellulose membranes (Amersham Biosciences, Slough, UK). After blocking, the membrane samples were incubated overnight at 4 °C with primary antibodies: NLRP3 (dilution 1:500, Santa Cruz Biotechnology, Santa Cruz, CA, USA), CASP-1 (dilution 1:500, Santa Cruz Biotechnology, CA, USA), IL-1β (dilution 1:500, Santa Cruz Biotechnology, CA, USA), P2X7 receptor (dilution 1:500, Santa Cruz Biotechnology, CA, USA), A1 receptor (dilution 1:500, Santa Cruz Biotechnology, CA, USA), TNF-α (dilution 1:500, Santa Cruz Biotechnology, CA, USA), IL-6 (dilution 1:500, Santa Cruz Biotechnology, CA, USA), and IL-10 (dilution 1:500, Santa Cruz Biotechnology, CA, USA). Afterward, anti-rabbit or anti-mouse secondary antibodies were incubated in these membranes (dilution 1:10.000, Santa Cruz Biotechnology) for 90 min at room temperature. The membranes were incubated with an enhanced chemifluorescent substrate (Amersham Biosciences) and analyzed with an Amersham Imager 600. The membranes were reprobed and tested for β-actin immunoreactivity as a control for the protein concentration. The band intensity was quantified via ImageJ 1.6.0 version to determine the relative protein density [116].

### 4.6. Statistical Analysis

The results were plotted in Microsoft Excel (Microsoft Office Professional Plus 2019 Version) and then converted to percentages relative to the negative control. One-way ANOVA was used to compare groups with a significance level of *p* < 0.05. Tukey’s post hoc test was applied to correct multiple comparisons with α = 0.05. Data were statistically analyzed in the GraphPad Prism 8.0 software (GraphPad Prism*^®^*, San Diego, CA, USA) and presented as the mean ± standard error of the mean (SEM). Comparations with *p* < 0.05 were considered statistically significant.

## 5. Conclusions

The present study confirmed the anti-neuroinflammatory action of RSV in a BV-2 cell line of microglia exposed to NLRP3 inflammasome activation conditions, highlighting the role of P2X7 and A1 in this context for the first time. We demonstrated the inhibitory ligation of RSV with the NLRP3 PYD domain by in silico methods. RSV preventive treatment can reduce pro-inflammatory NLRP3 and CASP-1 levels by decreasing P2X7 cellular receptors while increasing A1. Finally, RSV is a possible candidate for further investigations as a potential treatment against neuroinflammatory diseases, especially for those characterized by the exacerbated activation of the P2X7 purinergic receptor and NLRP3 inflammasome in their pathophysiology.

## 6. Limitations

This present study used in silico and in vitro methodologies and, for the first time, presents the integration of purinergic receptors (P2X7 and A1) and the NLRP3 inflammasome, using RSV as a possible treatment to control neuroinflammation. However, this study also has limitations. Here, we used a cell line obtained commercially. In this regard, using primary cultured microglia, cerebral organoids (3D culture), and in vivo evaluations could be promising alternatives to perform additional experiments to obtain similar and complementary results. Indeed, by considering more complex models mimicking a living organism, we could confirm and complement our findings.

Furthermore, this study did not evaluate the in vitro binding capacity of RSV with the PYD domain of NLRP3. However, the results suggest that RSV can act as an anti-inflammatory agent by regulating the NLRP3 mRNA levels and protein density. We also emphasize that our experimental analyses focused on the cytokines NLRP3 and CASP-1, which confirmed the action of RSV through priming signaling and the regulation of NLRP3 at the gene expression and protein density levels.

It is important to mention that this study did not directly analyze its biological potential in the NLRP3 assembly mechanism. On the other hand, our findings provide a potential reference for other research groups interested in analyzing this specific mechanism. We suggest that P2X7 acts as an induction mechanism for NLRP3 assembly, considering the elevated levels of the CASP-1 protein detected by the Western blot analysis (pro-caspase-1 cleaved to CASP-1 via inflammasome activation). In summary, RSV decreases the P2X7 purinergic receptor’s expression, and we believe that it reduces inflammasome assembly and releases pro-inflammatory markers, including CASP-1 and IL-1β. We strongly believe that our results are relevant and contribute to the scientific literature, bringing new perspectives regarding the mechanistic understanding of RSV’s anti-inflammatory effects through an innovative method of NLRP3 inflammasome activation, highlighting the P2X7 receptor’s role in this context.

## Figures and Tables

**Table 1:** Molecular docking between the PYD domain of NLRP3 and RSV or MCC950 ligands.

Ligand	Affinity (kcal/mol)	Estimated Ki	RMSD (Å)
RSV	−6.7	12.27 µM	0.480
MCC950	−8.4	696.25 nM	0.541

**Table 2:** Root mean square deviation (RMSD), radius of gyration (Rg), and solvent-accessible surface area (SASA) statistics for the APO, MCC950, and RSV systems, obtained throughout 100 ns molecular dynamics simulations.

Ligand	RMSD		SASA		Rg	
	Average (Å)	Standard Deviation (Å)	Average (Å^2^)	Standard Deviation (Å^2^)	Average (Å)	Standard Deviation (Å)
APO	2.26	0.33	5975.17	226.85	22.59	1.87
RSV	2.17	0.33	5751.66	231.06	21.55	1.22
MCC950	3.25	0.60	5802.91	209.53	27.03	2.18

**Table 3:** Binding free energy (ΔG_bind) components calculated by MM/GBSA for the complexes between the NLRP3 PYD domain and the ligands MCC950 and RSV. Mean values and standard deviations were obtained from 100 snapshots extracted from the last 10 ns of the simulation.

Molecule	∆E_vdW_	∆E_ele_	∆G_GB_	∆G_nonpol_	∆G_MM/GBSA_
RSV	−31.9652 ± 2.9491	−23.0579 ± 4.0545	37.3798 ± 3.1234	−4.7990 ± 0.2427	−22.4423 ± 5.9119
MCC950	−22.4536 ± 7.2380	−0.6858 ± 5.3272	12.4086 ± 6.4026	−2.9039 ± 1.0302	−13.6347 ± 11.0825

## Data Availability

The data presented in this study are available in this article. Further inquiries can be directed to the corresponding author.

## Supplementary Materials

The following supporting information can be downloaded at: https://www.mdpi.com/article/10.3390/ph18070950/s1, Supplementary Material S1: RSV cytotoxicity measurements in microglial cells (BV-2 cell line)—in vitro safety profile; Supplementary Material S2: Complete membranes corresponding to the Western blot presented in Figure 7 and Figure 8 of the article; Supplementary Material S3: Primers forward (F) and reverse (R) for gene expression.

## Abbreviations

The following abbreviations are used in this manuscript:

ASC	Apoptosis-associated adapter protein containing the CARD domain
CASP-1	Caspase-1
CNS	Central nervous system
DAMPs	Damage-associated patterns
DCFH-DA	2′-7′-Dichlorodihydrofluorescein diacetate
DMSO	Dimethylsulfoxide
dsDNA	Extracellular double-stranded DNA
FSB	Fetal bovine serum
FTSITE	Functional transformation SITE
IL-10	Interleukin-10
IL-18	Interleukin-18
IL-1β	Interleukin-1 beta
IL-6	Interleukin-6
LPS	Lipopolysaccharide
MM/GBSA	Generalized Born and surface area continuum solvation calculations
NIG	Nigericin
NLRP3	NOD-like receptor family pyrin domain containing 3
NO	Nitric oxide
NPT	Isothermal–isobaric ensemble
PAMPs	Pathogen-associated molecular patterns
PFA	Paraformaldehyde
PI	Propidium iodide
Rg	Radius of gyration
RMSD	Root mean square deviation
RNS	Reactive nitrogen species
ROS	Reactive oxygen species
RMSF	Root mean square fluctuation
RSV	Resveratrol
SASA	Solvent-accessible surface area
SNP	Sodium nitroprusside
TNF-α	Tumor necrosis factor-alpha
