# Supplementary material for: Resveratrol Alleviates Inflammatory Response Through P2X7/NLRP3 Signaling Pathway: In Silico and In Vitro Evidence from Activated Microglia
Source: Pharmaceuticals (Basel). 2025 Jun 24;18(7):950. doi: 10.3390/ph18070950 (PMC12298262; doi:10.3390/ph18070950)
Supplement: Supplementary file 1 [file pharmaceuticals-18-00950-s001.zip › pharmaceuticals-3683389-supplementary.pdf]

Supplementary Material S1 RSV cytotoxicity measurements in microglial cells (BV-2 cell line) – *in vitro* safety profile. (**a**) Cell viability; (**b**) dsDNA free content; (**c**) Nitrite levels; (**d**) ROS levels for 24 hours cells exposure to RSV; (**e**) Cell viability; (**f**) dsDNA free content; (**g**) Nitrite levels; (**h**) ROS levels for 48 hours cells exposure to RSV; (**i**) Cell viability; (**j**) dsDNA free content; (**k**) Nitrite levels; (**l**) ROS levels for 72 hours cells exposure to RSV. SNP- Sodium nitroprusside. The experiments were performed in triplicate. The statistical analysis was conducted by one-way ANOVA following by Tukey post hoc test. Data are expressed as mean values ± SEM. *P < 0.05; **P < 0.01; ***P < 0.001; ****P < 0.0001. *means comparison to the control group.

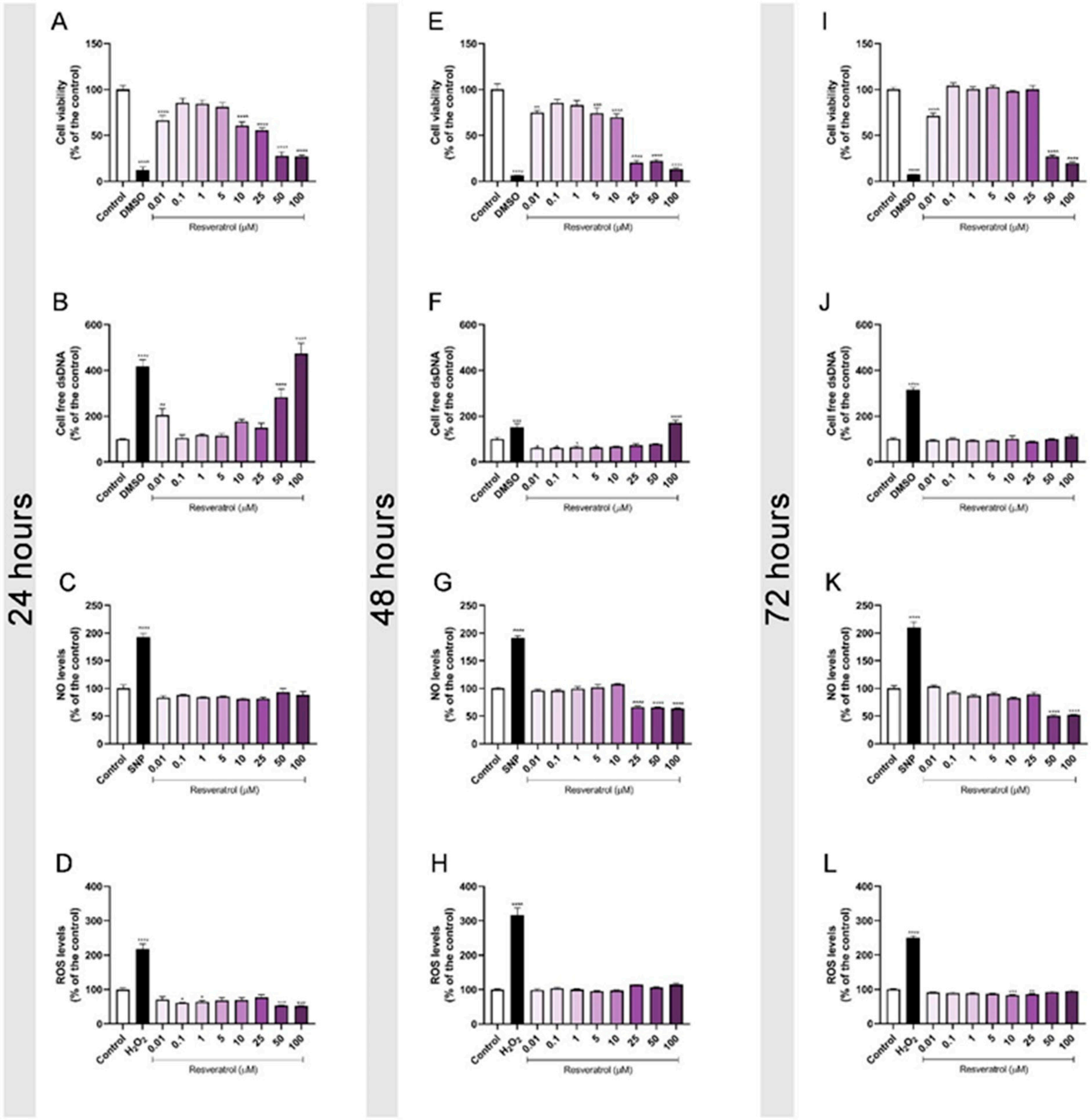

Supplementary Material S2 Complete membranes corresponding to the Western blot presented in Figures 7 and 8 of the article. The membranes show the detection of the density of the proteins NLRP3 (**a**), caspase-1 (**b**), IL-1β (**c**), IL-6 (**d**), TNF-α (**e**), IL-10 (**f**), P2X7 (**g**), A1 (**h**) and beta-actin (**i** and **j**) in three experimental replicates. The bands of interest are detected consistently at the expected molecular weight. Nonspecific bands are present, but do not compromise the interpretation of the results. Some membranes were subjected to the stripping and reprobing process, which may justify background variations or absence of labeling for beta-actin in certain replicates. Protein loading was normalized based on total protein quantification.

A

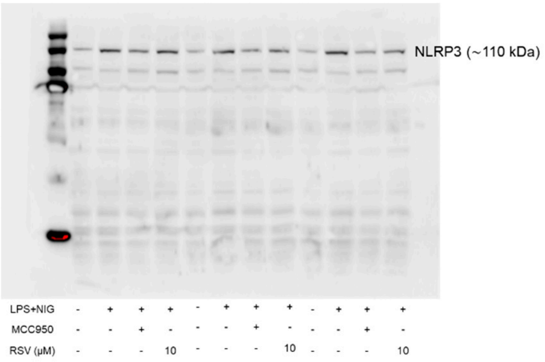

B

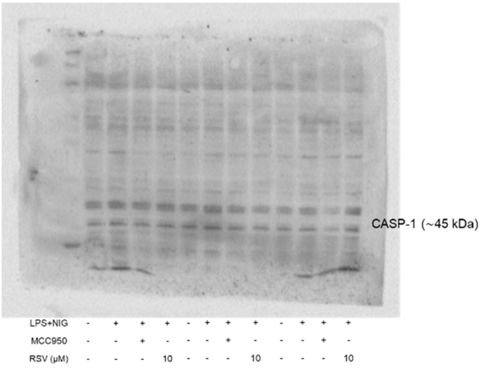

C

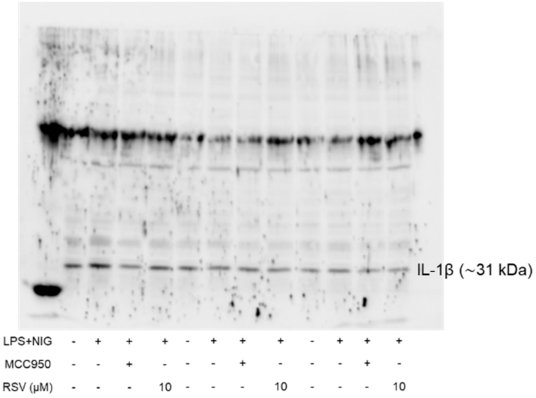

D

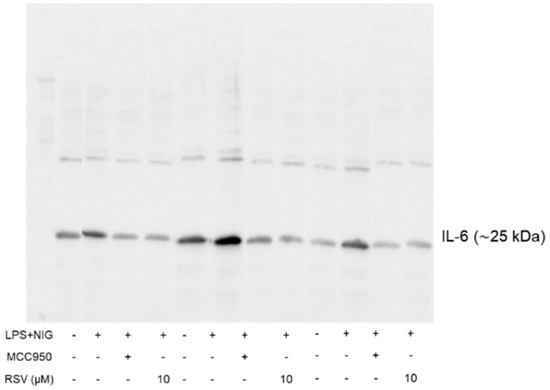

E

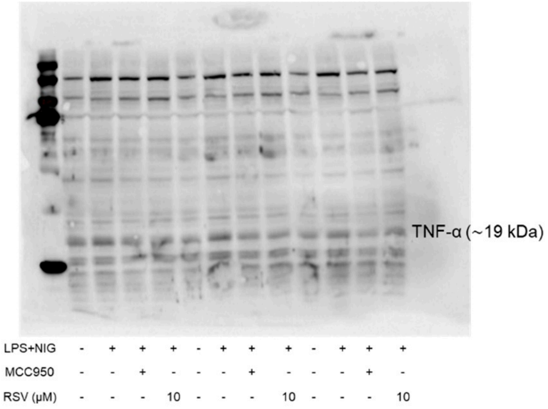

F

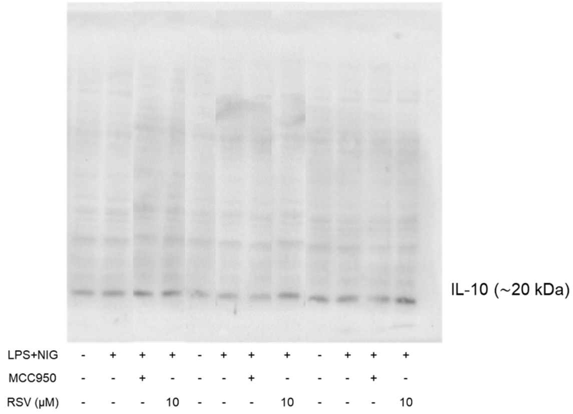

G

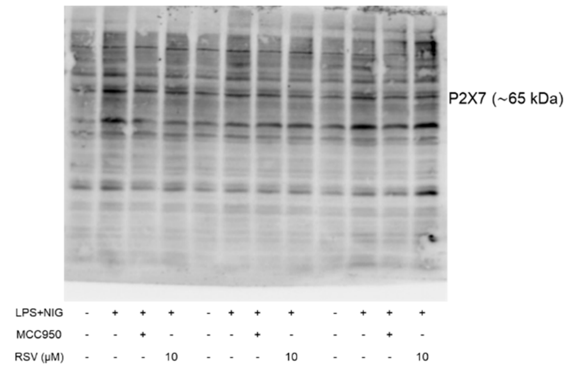

P2X7 (~65 kDa)
LPS+NIG - + + + - + + + - + + +
MCC950 - - + - - - + - - - + -
RSV (μM) - - - 10 - - - 10 - - - 10

H

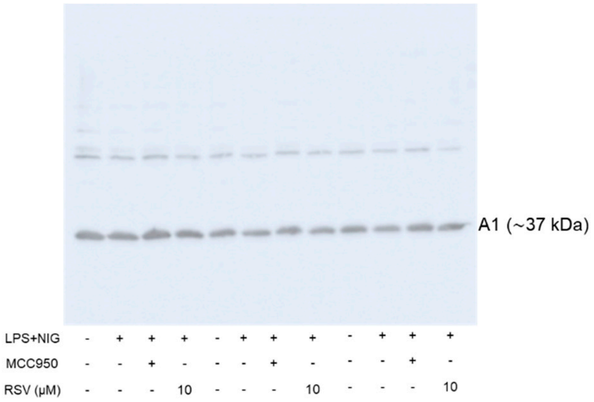

A1 (~37 kDa)
LPS+NIG - + + + - + + + - + + +
MCC950 - - + - - - + - - - + -
RSV (μM) - - - 10 - - - 10 - - - 10

I

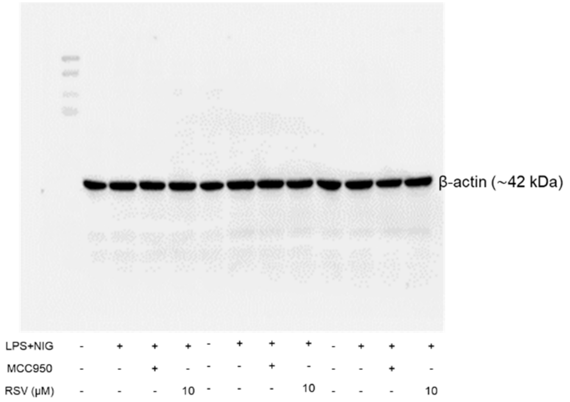

β-actin (~42 kDa)
LPS+NIG - + + + - + + + - + + +
MCC950 - - + - - - + - - - + -
RSV (μM) - - - 10 - - - 10 - - - 10

J

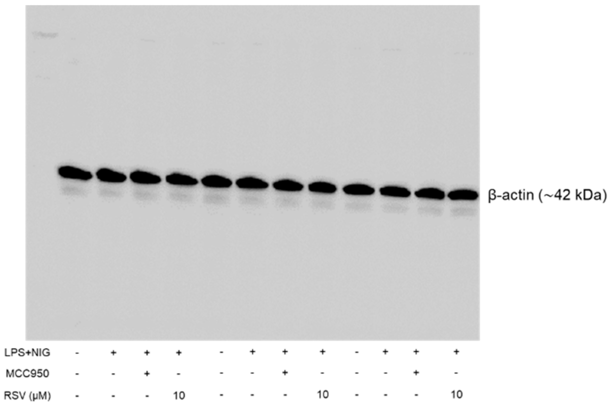

β-actin (~42 kDa)
LPS+NIG - + + + - + + + - + + +
MCC950 - - + - - - + - - - + -
RSV (μM) - - - 10 - - - 10 - - - 10

Supplementary Material S3 Primers forward (F) and reverse (R) for gene expression.

Gene	Sequence (5'-3')	Blast code
**IL-1β**	F – GGTACATCAGCACCTCACAA<br>R – TAGAAACAGTCCAGCCCATAC	NM_008361.4
**IL-6**	F – CTTCCATCCAGTTGCCTTCT<br>R – CTCCGACTTGTGAAGTGGTATAG	X54542.1
**IL-10**	F – ACAGCCGGGAAGACAATAAC<br>R – CAGCTGGTCCTTTGTTTGAAAG	NM_010548.2
**TNF-α**	F – TTGCTCTGTGAAGGGAATGG<br>R – GCTCTGAGGAGTAGACAATAAAG	X02611.1
**NLRP3**	F – CCATACCTTCAGTCTTGTCTTC<br>R – CTGCCACAAACCTTCCATCTA	NM_145827.4
**CASP-1**	F – GGCACATTTCCAGGACTGACTG<br>R – GCAAGACGTGTACGAGTGGTTG	NM_009807.2
**β-Actin**	F – CCGTAAAGACCTCTATGCCAAC<br>R – AGGAGCCAGAGCAGTAATCT	NM_007393.5
